# Home-Based Intervention Program to Reduce Food Insecurity in Elderly Populations Using a TV App: Study Protocol of the Randomized Controlled Trial Saúde.Come Senior

**DOI:** 10.2196/resprot.6626

**Published:** 2017-03-13

**Authors:** Ana Maria Rodrigues, Maria João Gregório, Pierre Gein, Mónica Eusébio, Maria José Santos, Rute Dinis de Sousa, Pedro S Coelho, Jorge M Mendes, Pedro Graça, Pedro Oliveira, Jaime C Branco, Helena Canhão

**Affiliations:** ^1^ EpiDoC Unit, Centro de Estudos de Doenças Crónicas NOVA Medical School, Universidade Nova de Lisboa Lisboa Portugal; ^2^ Sociedade Portuguesa de Reumatologia Lisboa Portugal; ^3^ Instituto de Medicina Molecular Rheumatology Research Unit Lisboa Portugal; ^4^ Faculdade de Ciências da Nutrição e Alimentação Universidade do Porto Porto Portugal; ^5^ Hospital Garcia de Orta Almada Portugal; ^6^ NOVA Information Management School Universidade Nova de Lisboa Lisboa Portugal; ^7^ Programa Nacional para a Promoção da Alimentação Saudável Direção-Geral da Saúde Lisboa Portugal; ^8^ Católica-Lisbon School of Business and Economics Universidade Católica Portuguesa Lisboa Portugal; ^9^ Serviço de Reumatologia do Hospital Egas Moniz Centro Hospitalar Lisboa Ocidental Lisboa Portugal

**Keywords:** information and communication technology, new technologies, TV app, healthy lifestyle promotion, food insecurity

## Abstract

**Background:**

The limited or uncertain access to adequate food in elderly people includes not only economic restrictions but also inability of food utilization due to functional or cognitive impairment, health problems, and illiteracy.

**Objective:**

The aim of this work is to present the protocol of the randomized controlled trial Saúde.Come Senior, an educational and motivational television (TV)-based intervention to promote healthy lifestyles and decrease food insecurity in elderly people.

**Methods:**

A randomized controlled study will be conducted in subjects aged 60 years and older with food insecurity, identified at 17 primary care centers in the Lisboa e Vale do Tejo health region in Lisbon, Portugal. The primary outcome will be the changes in participants’ food insecurity score (evaluated by the Household Food Insecurity Scale) at 3 months. Change in other outcomes will be assessed (dietary habits, nutritional status, physical activity, health status, and clinical outcomes). Subjects will be followed over 6 months; the intervention will last 3 months. Data collection will be performed at 3 different time points (baseline, end of intervention at 3 months, and follow-up at 6 months). The intervention is based on an interactive TV app with an educational and motivational program specifically developed for the elderly that has weekly themes and includes daily content in video format: (1) nutrition and diet tips for healthy eating, (2) healthy, easy to cook and low-cost recipes, and (3) physical exercise programs. Furthermore, brief reminders on health behaviors will also be broadcasted through the TV app. The total duration of the study will be 6 months. The intervention is considered to be effective and meaningful if 50% of the individuals in the experimental group have a decrease of 1 point in the food insecurity score, all the remaining being unchanged. We expect to include and randomize 282 (141 experimental and 141 control) elderly with food insecurity. We will recruit a total of 1,128 subjects considering that 50% of the target individuals are food insecure (based on INFOFAMÍLIA Survey) (567) and about 50% of those will adhere to the study (282).

**Results:**

The randomized controlled trial with the 12-week home-based intervention with a comprehensive program on healthy eating and physical activity delivery is planned to start recruiting participants at the end of 2017.

**Conclusions:**

This study will assess the efficacy of this innovative tool (Saúde.Come Senior) for disseminating relevant health information, modifying behaviors, and decreasing food insecurity in an easy, low-cost, and massive way.

## Introduction

The world is facing a situation without precedent: soon there will be more older people than children and more people at extreme old ages than ever before [1]. In fact, by 2025 more than 20% of Europeans will be aged 65 years or over, with a particularly rapid increase in the number of over 80s [1]. Portugal has a high proportion of elderly persons and is one of the European countries with the lowest birth rate [2]. As both the proportion of older people and the length of life increase throughout the world, some key questions arise. Will population aging be accompanied by a longer period of good health, a sustained sense of well-being, and extended periods of social engagement and productivity, or will it be associated with more illness, disability, and dependency? What are the consequences of the European economic distress on the health of the elderly?

The impact of an economic crisis may be particularly acute for older people, mainly for those who are physically vulnerable, living in poverty, or dependent on private pensions, leading to adverse lifestyles and health outcomes. Adverse lifestyles and health outcomes include increasing food insecurity; use of tobacco, alcohol, and drugs; increasing depression and anxiety; and a general neglect of overall health [3]. In fact, elderly people with food insecurity report reduced quality, variety, or desirability of diet which leads to higher morbidity through decompensated chronic noncommunicable diseases (diabetes mellitus, hypertension, dyslipidemia), low muscle strength with less mobility, higher mortality, and higher health care costs [4,5]. Many have significant cognitive, psychiatric, and physical problems yet do not seek assistance. Assessment and intervention in these cases requires an interdisciplinary approach. An understanding of risk factors, the clinical evaluation process, competency issues, and basic management strategies is integral to good care.

According to the internationally recognized definition, “food security is the situation that exists when all people, at all times, have physical and economic access to sufficient safe and nutritious food that meets their dietary needs and food preferences for an active and healthy life” [6]. In contrast, food insecurity is a broad concept that includes attributes related with the uncertainty or worry about food, inadequate quality of food, inadequate quantity of food, food acquired through socially unacceptable means, and lack of consistent access to adequate food [7]. Food insecurity is often associated with economic constraints. The rates of food insecurity have been rising worldwide [7]. While the United States is one of the wealthiest nations in the world with a rich and abundant supply of food and resources, 14.3% of US households were food insecure at some point during 2013 [8]. A study of Portuguese primary care center attendees in 2013 showed that 50.7% were food insecure [9]. Furthermore, more than one-third of Portuguese adults with food insecurity are overweight (41.0% in moderate food insecurity and 37.7% in severe food insecurity categories) [10]. This situation needs particular attention if we consider that Portugal is one of the Organization for Economic Cooperation and Development countries with highest levels of income inequalities [11].

Food insecurity has been shown to be associated with poor self-rated health and several noncommunicable diseases such as diabetes, hypertension, fibromyalgia, and osteoporosis [12,13].

In general, food insecurity has been less well studied in the elderly. Data from the United States suggested that food insecurity decreases as age increases. The prevalence of food insecurity was 20.4% among US adults aged between 40 to 49 years and 15.7% among older adults aged between 60 to 69 years [14]. The same trend was observed for Canada: the prevalence of food insecurity was lower in elderly people (16% vs 29% for the total sample) [15]. However, the nutrition and health consequences might be potentially more severe for older adults. In elderly populations, food insecurity might occur as a result of other factors rather than constraints of financial resources. There is evidence that food insecurity among the elderly can be associated with health or mobility problems (functional impairment), which can also compromise the access to healthy food.

Indeed, the concept of food insecurity in elderly persons also includes nutritional deficits and other relevant aspects related with limited or inability to use food due to functional impairments and health problems [16,17]. For older adults, who generally require special attention for optimal nutrition, food insecurity has been a risk factor for poor nutritional status and low muscle power especially in those with physical disabilities, increasing hospitalizations and death [4,5].

Information and communication technology (ICT) solutions can be used as a personalized cost-effective smart model of health empowering subjects to take more responsibility for their own health and quality of life. Moreover, ICTs have the potential to make a major contribution to improve access to quality services while controlling costs. Elderly people spend a significant amount of time watching television (TV) [18], which can be used as a tool to improve health literacy and a vehicle for promoting health lifestyles. Promoting healthy lifestyles in elderly populations is crucial to slow physical decline and improve well-being, delay the deterioration of health, and reduce the risk of mortality [19].

For this study, we hypothesized that a home-based intervention program on dietary and physical activity through a TV app will improve food security as well as clinical endpoints such as nutritional status, body composition, balance, strength, and quality of life in the elderly population. Considering the broad definition of the food insecurity concept and its 4 main dimensions (availability of food, physical and economic access to food, food utilization, and stability of the other dimensions) [20], our intervention program will be focused on the dimension of food security related to the use or utilization of food, which might be affected by literacy in food and health. Our hypothesis is that a TV-based intervention might have a large potential in promoting healthy lifestyles among low socioeconomic status groups and for people with low literacy, since TV is the most frequently used ICT by low literacy people.

This paper describes the study protocol of a home-based randomized controlled trial aimed at evaluating the impact of a TV-based intervention on healthy lifestyle promotion in elderly subjects in food insecurity reduction.

## Methods

### Design Overview

**Figure 1:**
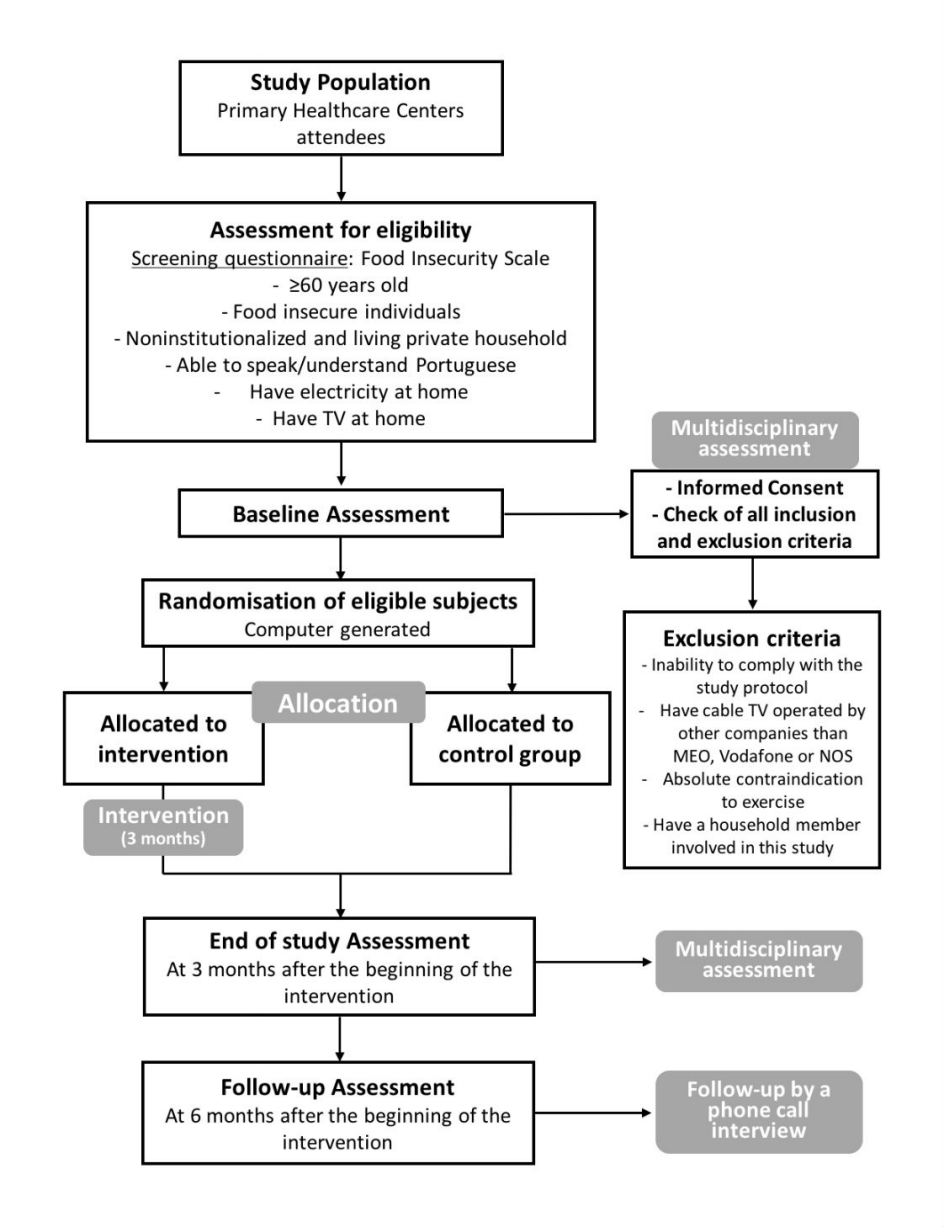
Study design flowchart.

This is a randomized controlled study based on an intervention program on diet and physical activity that uses a TV app in about 282 elderly subjects aged 60 years and older with food insecurity identified at 17 primary care centers in the *Lisboa e Vale do Tejo* health region in Portugal. Subjects will be followed over 6 months after the beginning of intervention, which will last 3 months. The research protocol of this study is illustrated in the study design flowchart depicted in Figure 1.

### Study Population and Recruitment Plan

A convenience sample of food-insecure elderly people from 17 primary care centers of *Lisboa e Vale do Tejo* health region will be enrolled in this study. All elderly subjects who attend health services in the selected primary care centers during the recruitment period will be approached by our research team and asked to complete a questionnaire for the initial screening. The questionnaire includes a food insecurity scale to identify food-insecure elderly persons, and an authorization request form will be provided to obtain consent for a further contact. All elderly subjects identified as food insecure will be invited to participate in this study. The baseline assessment will be performed during an appointment at the primary care center with a multidisciplinary team that includes a medical doctor, nurse, nutritionist, and physiotherapist. The medical doctor will ask for informed consent and check inclusion and exclusion criteria.

### Eligibility

To be eligible for this randomized controlled study, subjects must fulfill all of the following inclusion criteria: (1) aged 60 years or older, (2) identified as food insecure in the recruitment [21], (3) able and willing to give written informed consent and phone number contact and comply with other requirements of the study protocol, (4) Portuguese speaker or able to understand Portuguese, (5) noninstitutionalized and living in a private household in Portugal, (6) have electricity at home, and (7) have cable TV with box at home. The exclusion criteria are as follows: (1) inability to comply with study protocol (hearing/visual loss, cognitive impairment, etc); (2) absolute contraindication to exercise; (3) have cable TV operated by other companies than MEO, Vodafone, or NOS; and (4) have a household member involved in this study.

### Description of the Intervention

Our intervention program is based on a cognitive behavioral strategy for promoting lifestyle behavior changes, combining sets of different strategies that will be important to the learning process as well as to behavioral changes.

This tool was developed based on the transtheoretical model [22] that conceptualizes the process of intentional behavior change in 5 stages, from precontemplation to maintenance. This intervention program accompanies the participants through all these phases. At T0 clinical appointments, doctors, nutritionists, physiotherapists, and nurses lead the participant through the stages of precontemplation and contemplation. In the preparation and action phases—when the subject intends to change behaviors and in fact does change them—the intervention program offers them all the cognitive and behavioral tools to achieve the changes. In addition, the intervention promotes helping relationships, since participants have at their disposal the telephone number and the possibility to contact the team as regularly and as often as they wish. The program also considers the phases of maintenance and prevention of relapse in the T1 clinical appointment and 3 months after the program.

The contents of the intervention program using a TV app were developed closely regarding Kolb’s experiential learning model [23,24]: videos on physical exercise and recipes (reflective and active experimentation); videos with tips on nutrition and healthy lifestyles (abstract conceptualization); queries; reminders; and the option to contact the medical doctor, nutritionist, nurse, and physiotherapist.

We designed an interventional program using an interactive TV app to improve dietary habits and physical activity levels among elderly populations with food insecurity. Of the several ICTs available, we have chosen the TV app because the majority of elderly people with food insecurity watch TV [25]. Unpublished data from our group have shown that TV is the most important source of health information for this group.

Our intervention consists of a 12-week home-based intervention with a comprehensive program on healthy eating and physical activity, specially designed for the elderly. This program is an innovative interactive TV app for healthy lifestyle promotion and was designed taking into account (1) the need to educate elderly people about the importance of a healthy diet and exercise, (2) the need to explain that low household income is not a barrier to healthy lifestyles and it is possible to have a healthy diet and physical activity habits at low cost, and (3) the importance of motivating individuals to eat healthier and engage in more physical activity in order to reduce noncommunicable diseases. This interventional program is composed of 3 parts: nutrition and diet tips for healthy eating, low-cost healthy recipes, and physical exercise programs. The nutrition tips content was adapted for people who have economic difficulties in accessing food. In fact, all of the contents were based on a book published by the Portuguese Directorate-General of Health, developed with the main aim of improving dietary habits of low-income people. For example, we promote the consumption of eggs as one of the most economical choices among the good sources of protein, we promote the consumption of vegetarian sources of protein (pulses), and we promote the consumption of seasonal fruit and vegetables because fresh produce often costs less and is a nutritious option. The recipes were developed in order to show how to prepare healthy, tasty, and low-cost recipes. Finally, the physical activity video shows that is possible to do some physical activity at home without spending money.

All of the contents were disseminated on a dedicated channel through a TV app in a video format. As part of the motivation approach of our intervention program, brief reminders on health behaviors will also be diffused weekly through the TV app. These brief reminders will be powerful in recalling and stimulating study participants to adhere, encouraging lifestyle changes. Moreover, frequent contacts with participants are a relevant aspect of successful behavior change intervention programs [26], and follow-up monitoring is included in our program in order to monitor changes in specific behaviors and knowledge during the intervention period. With that purpose, short questionnaires will be delivered weekly through the TV. Thus, the interactive app will be used to collect data (short questionnaires) to evaluate the program compliance and learning. With these short questionnaires we aim to capture lifestyle changes and evaluate the learning along the intervention period. These questionnaires can easily be answered by pushing TV remote control buttons.

This 12-week TV-delivered program is composed by thematic weeks, and all of the contents (“nutrition and diet tips for healthy eating” videos, “healthy recipes” videos, brief reminders, and short questionnaires) developed for each week were specially designed to take into account the thematic week (Table 1).

**Table 1:** Theme of each week of the interventional TV app on healthy eating and physical activity.

Week	Theme	Content
1	**Vegetable week**	
		Health benefits of vegetable consumption
		Recommendations for vegetable intake
		How to increase the daily intake of vegetables
2	**Water week**	
		Water, hydration, and health
		Recommendations for water intake
		Food resources that contain water
3	**Milk week**	
		Health benefits of consumption of milk and other dairy products
		Recommendations for milk intake
4	**Olive oil week**	
		Olive oil as a healthy cooking oil option
		Adequate amounts for using olive oil for food preparation and cooking
5	**Fruit week**	
		Health benefits of fruit consumption
		Recommendations for fruit intake
6	**Salt week**	
		Health risks of salt intake
		Recommendations for salt intake
		Foods with high and low salt content
		Strategies to reduce salt intake
7	**Meat, seafood, and eggs week**	
		Importance of consuming adequate amounts of meat, seafood, and eggs
		Adequate portions of meat, seafood, and eggs
		Healthier food options within the different foods of this group
8	**Vegetable soup week**	
		Health benefits of vegetable soup consumption
		Standard recipe for a healthy vegetable soup
9	**Vegetable and fruit week**	
		Health benefits of vegetable and fruit consumption
		Recommendations for vegetable and fruit intake
		Meeting vegetable and fruit intake recommendations
		How choose the low cost options for these foods
10	**Healthy cooking week**	
		How to cook healthy food
11	**Sugar week**	
		Health risks of sugar intake
		Recommendations for sugar intake
		Foods with high and low sugar content
12	**Pulses week**	
		Health benefits of pulses consumption
		Recommendations for pulses intake
		How to include pulses in your diet daily intake

The themes for each week and consequently the contents of each video on nutrition and diet tips for healthy eating were chosen considering the main concerns on diet, in particular the main risk factors for noncommunicable diseases that are associated with unhealthy diets and the nutritional requirements for elderly people [27-29]. The low-cost healthy recipes are chef-created recipes and were specifically developed for our program by a popular Portuguese TV chef with a consulting nutritionist. The physical activity program was developed by physical exercise experts with the main goal of encouraging at least 30 minutes of physical exercise at home 3 times per week [30].

The delivery of these contents through the TV app will be scheduled for specific days of each week in order to develop a routine and constant program over the intervention period. Each day, 1 program will be available (Table 2). The TV app was developed by an external company, and the main TV cable operators agreed to disseminate this app.

**Table 2:** Weekly schedule of the intervention delivery.

Weekday	Hour	Program delivery
Monday	2:00 PM	Nutrition and diet tips for healthy eating
Tuesday	12:00 PM	Healthy and low-cost recipes
Wednesday	9:00 AM	Physical exercise program
Thursday	2:00 PM	Brief reminder
Friday	Not fixed	Questionnaire
Saturday	Not fixed	Questionnaire
Sunday	—	—

During the development of the TV app, a focus group was conducted by a trained psychologist using a semistructured interview to assess the concept of the TV app, its usability, the adherence intention, and the expected impact on behavior modification. To this end, 11 subjects (6 women and 5 men) with similar characteristics to the study population were enrolled. In terms of the concept of a “TV-based intervention on healthy lifestyles promotion in the reduction of food insecurity in elderly subjects,” the members of the focus group generated immediate, very positive, and homogeneous reactions in respondents. The unsolicited comments were many and denoted a strong reception of the concept by all respondents. On the other hand, there was a clear projective identification with the tested app, since it addresses some of the issues that most concern this group: healthy lifestyles, diet, nutrition, and exercise.

Regarding the sense of usability, participants pointed out that the TV is a familiar device and, for that reason, considered the TV app easy to use. On the other hand, little sensitivity in the fingers to handle the remote control and the lack of vision to distinguish a few keys and buttons were stated as likely barriers to its use. Concerning the adherence intention, all respondents were willing to have this app in their homes and report feeling motivated to use it because it is a targeted tool for them.

The TV app seemed to have the potential to generate a positive impact on changing behaviors of seniors. This focus group reported general satisfaction and motivation. Nevertheless, the impact assessment will only be possible at a later stage of use. By using this cognitive behavioral strategy, we reinforce elderly learning and behavioral modifications for promoting lifestyle behavior changes.

**Figure 2:**
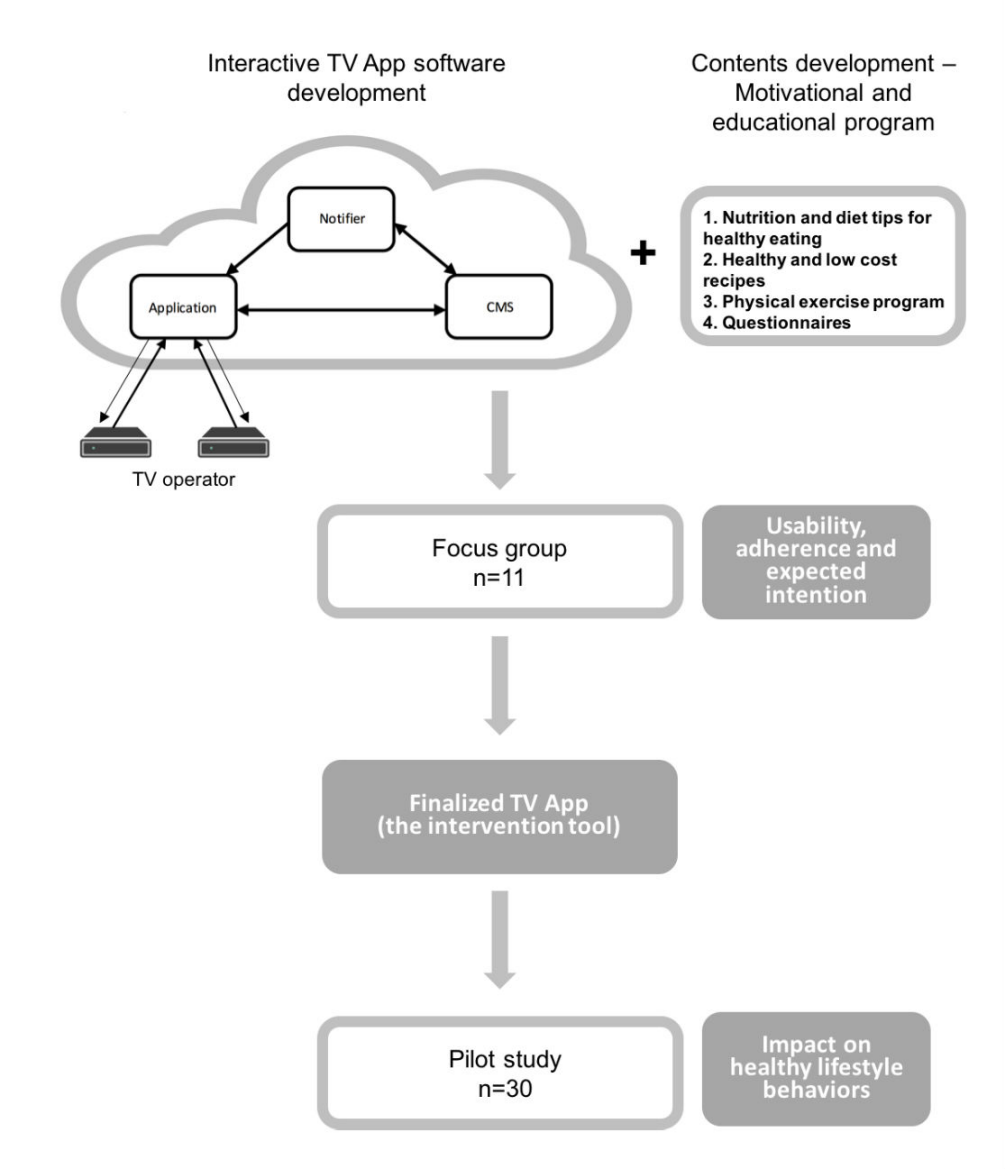
Television app development process.

After the TV app is completed, a pilot study will be performed in order to test (1) the TV app diffusion conditions by the TV cable operators, (2) TV app user experience, (3) subjects’ adherence to the TV app, and (4) subjects’ behavioral changes through qualitative analysis. For this study a small sample (n=30) of elderly people with and without food insecurity will be recruited (Figure 2).

### Data Collection

During the study, 3 different collection points are planned: baseline assessment, end of intervention assessment (at 3 months after the beginning of the intervention) and follow-up assessment (at 6 months after the beginning of the intervention). Clinical assessments will be performed by a multidisciplinary team (medical doctor, nurse, nutritionist, and physiotherapist) at primary care centers involved in this study. Structured evaluations will be conducted with a computer-assisted personal interview (CAPI) system. Each assessment will start with the medical doctor evaluation, followed by the nutritionist, physiotherapist, and nurse evaluation. As explained above, before the baseline assessment, the medical doctor will obtain the informed consent and check the eligibility of the participant.

During the baseline assessment, the following sociodemographic data and socioeconomic profile variables will be recorded: age, sex, ethnicity, education, marital status, measures of wealth (used to generate income quintiles), and household income. Also, medical history and physical examination will be performed by the medical doctor. In addition, the following data will be collected: health care resource consumption (number and type of outpatient clinic visits, hospitalizations, home care assistance, and other needs for health care services in the previous 12 months), health-related quality of life (European Quality of Life questionnaire with five dimensions and three levels [EQ-5D-3L] and the Functional Assessment of Chronic Illness Therapy–Fatigue [FACIT-F]), lifestyle behaviors (alcohol and coffee consumption, smoking habits, and regular physical activity), physical function (Health Assessment Questionnaire [HAQ]), anxiety and depression symptoms (Hospital Anxiety and Depression Scale [HADS]), self-reported chronic diseases (high cholesterol level, high blood pressure, allergy, gastrointestinal disease, mental disease, cardiac disease, thyroid and parathyroid disease, urolithiasis, pulmonary disease, hyperuricemia, malignancy, neurologic disease, and rheumatic and musculoskeletal diseases), and information regarding pharmacological and nonpharmacological therapies.

A nutrition and physiotherapy assessment will be performed at baseline and end of intervention assessment (at 3 months). The nutritionist will collect data regarding food insecurity (Household Food Insecurity Scale [HFIS] adapted from the US Department of Agriculture Household Food Security Survey Module [21]), dietary habits (2-day 24-hour diet recall, Prevención con Dieta Mediterránea [PREDIMED] index [31], and food frequency questions), anthropometric data (height, weight, and waist circumference), body composition data obtained by bioimpedance (fat mass, fat-free mass, total body water), attitudes and barriers on diet, food expenses, and other resources for food. Regarding the dietary habits assessment, a second 24-hour diet recall will be performed by phone call interview performed within 1 week.

Physiotherapy assessment will be conducted aiming at collect data regarding physical activity (Elderly Mobility Scale [EMS] [32] and International Physical Activity Questionnaire [IPAQ]) and muscle strength (hand grip, knee extension, and hip flexion by using a dynamometer). The assessment scales used in the study are all Portuguese-validated versions, and the average time for the evaluation will be 90 minutes.

The nurse will perform a structured questionnaire in order to capture baseline knowledge on healthy lifestyles. This questionnaire will be repeated during the 3-month and 6-month follow-up assessments.

Blood samples will also be collected in order to obtain data regarding the nutritional status and serological biomarkers of cardiovascular risk (albumin and prealbumin levels, insulin resistance, cholesterol levels, hemoglobin A_1c_, adipocytokines, high sensitivity C-reactive protein, interleukin 1, interleukin 6, and tumor necrosis factor).

At the 6-month follow-up time point, the assessment will be telephonically performed with the assistance of a CATI system (an in-house software platform) by a team of research assistants. These data will be collected in a standardized form; database access is protected by unique username and password for each research team member. In this telephone interview, we will collect data regarding food insecurity (HFIS [21]), dietary habits (2-day 24-hour diet recall, PREDIMED index [31], health-related quality of life and health status (E-5D-3L and FACIT-F), physical activity (IPAQ), consumption of health care resources (inpatient admissions and outpatient appointments), functional disability (HAQ), anxiety and depression symptoms (HADS), and lifestyle behaviors (see Multimedia Appendix 1 for a schedule of all collected data). Finally, the intervention group data regarding the adherence to the program will be monitored using the TV interactive data.

### Outcome Measures

The primary outcome assessed will be the changes in participant food insecurity score (evaluated by the HFIS) from baseline to 3 months. Secondary outcomes at 3 months will be changes in dietary habits (evaluated by 2-day 24-hour recall, PREDIMED index [31], and food frequency questions), changes in indicators of nutritional status (body mass index [BMI] and albumin and prealbumin levels), changes in muscle strength improvement (evaluated by dynamometer), changes in quality of life (EQ-5D-3L) [33], changes in fatigue scale (FACIT-F) [34], changes in serological markers of cardiovascular risk (insulin resistance, cholesterol levels, hemoglobin A_1c_, high sensitivity C-reactive protein, adipocytokines, interleukin 1, interleukin 6, and tumor necrosis factor), changes in physical activity (EMS [32] and IPAQ [35]); changes in body composition balance evaluated by bioimpedance, changes in inpatient admissions and outpatient appointments, changes in falls, and changes in knowledge regarding healthy lifestyle.

The secondary outcomes at 6 months will be changes in participant food insecurity score, changes in dietary habits (2-day 24-hour recall, PREDIMED index [31], and food frequency questions), changes in quality of life (EQ-5D-3L) [33], changes in fatigue scale (FACIT-F), changes in physical activity (IPAQ) [35], changes in inpatient admissions and outpatient appointments, changes in falls, and changes in knowledge regarding healthy lifestyle.

### Sample Size

Sample size was estimated considering a *t* test (independent samples) for comparing the mean change in food insecurity score from baseline to the end of study between group 1 (intervention) and group 2 (control). The intervention is considered to be meaningful if 50% of the individuals in group 1 have a decrease of 1 point in the food insecurity score, all the remaining being unchanged. This results in a difference of –0.5 between the mean changes of the 2 groups. Standard deviation of the change is assumed to be 1.5. Power is set at 0.80 and α=.05. We expect to include and randomize 141 experimental and 141 control elderly people with food insecurity (n=282) equally distributed among the 17 primary care centers. We are prepared to recruit around 70 individuals in each primary care center (a total of 1128 subjects are needed) because we assume that 50% (based on the INFOFAMÍLIA [National Survey on Food Insecurity in Portugal] survey) [36] of the target individuals are food insecure and that 50% of those will adhere to the study.

Of note, the standard approach to sample size calculation based on a clinically important difference was not possible to use in this study because data on interventions in food insecurity are scarce. We have considered the referred effect size based on the INFOFAMÍLIA survey data. In this study, the majority of Portuguese food-insecure households are in low food insecurity level (39.8%), and about 80% of those in the low food insecurity level obtained just 1 point in the food insecurity score, which means that if our intervention decreases the food insecurity score by 1 point we will be able to see a change from the low food insecurity level to the food security level.

### Randomization, Allocation, and Blinding

Randomization will be computer generated. Randomization will be stratified according to gender and food insecurity level (low/moderate vs severe) with permuted blocks of six. Subjects will be allocated to the study group versus control with a probability of 1:1.

We will ensure researchers taking follow-up outcome measures will be blinded to group allocation; after randomization, the intervention group will be informed that they will have access to the intervention program (TV app) by an independent assessor (single-blind).

### Data Analysis

Analysis will be performed with Stata software (StataCorp LLC). Continuous variables will be reported as mean and standard deviation (or in case of nonnormal distribution as median and interquartile range). Categorical variables will be displayed as frequencies or proportions. The association between food security status, dietary data, and health outcomes will be assessed through chi-square analysis of baseline data.

To assess the effect of the interventional program on food security, dietary intake, nutritional status and body composition, quality of life, muscle strength, fatigue scale, physical activity habits, and serological markers of cardiovascular risk, 2-tailed *t* tests will be used to compare the amount of change within each group (baseline, end of study [at 3 months] and follow-up [at 6 months]). Independent sample *t* tests will be used to compare the intervention group and control group on the same outcomes.

Dietary intake assessed with 2-day 24-hour recall will be analyzed according to the recommended number of servings from each food group of the Portuguese Food Wheel [37]. Intakes of micronutrients that tend to be low in vulnerable population groups (iron, calcium, folate, and vitamins A, C, and D) will be also analyzed. The percentage of individuals meeting the Estimated Average Requirement or Adequate Intake for iron, calcium, folate, and vitamins A, C, and D will be calculated [38]. Food Processor Nutrition Analysis software (ESHA Research) will be used to estimate the intakes of energy, macro-, and micronutrients.

The association between each outcome and the candidate covariables will be performed using generalized models. We will use multivariate logistic and linear (according to the outcome) regression models. The candidate covariates will be first analyzed by univariate regression. Covariates will enter the multivariate models if their *P* value is less than .25 in univariate analysis or considered clinically relevant in this setting. The selection of covariates will be stepwise by backward selection, according to the level of significance (<.05). Multilevel analysis and sensitive analysis will be performed when appropriate.

### Ethical Considerations

Study design was performed according to the principles established by the Declaration of Helsinki. The protocol was reviewed and approved by the NOVA Medical School Ethics Committee, by the National Committee for Data Protection (*Comissão Nacional de Proteção de Dados*), and by the Ethical Committee of the Regional Health Authority of *Lisboa e Vale do Tejo.*

The details of the protocol, including the study aims, methods, procedures, and measurements performed during the study, will be provided in written format and discussed with each potential subject. Written informed consent will be obtained for all subjects before any project-related procedure is performed. A copy of the signed and dated consent form will be given to the subject. Participants will also be informed about ethical issues such as confidentiality, the right to ask any questions during the study, and their right to withdraw at any time. The risks associated with participation in this study are minimal; they do not constitute a threat to confidentiality and are not expected to harm the participants.

Data protection is assured by a data encryption process, ensuring the confidentiality and anonymity of each study subject. Decryption is possible with a secure password known only to the principal investigator.

During the multidisciplinary assessments (baseline and end of study assessment), all participants with a new diagnosis of a chronic disease will be referred to their primary care physician for follow-up. Participants will receive laboratory test results by letter. If a clinically significant abnormality was depicted in the laboratorial results, the participant will also be advised to see his or her doctor for further investigation.

## Results

The present project was granted by the Public Health Initiatives Programme (PT06) and financed by European Economic Area (EEA) Grants Financial Mechanism 2009-2014. The pilot study with 30 elderly people to test user adherence, TV app diffusion conditions, and subjects’ behavioral changes through qualitative analysis has already been performed; results are being analyzed and the TV app is being adjusted according to the participants suggestions.

The randomized controlled trial with the 12-week home-based intervention with a comprehensive program on healthy eating and physical activity delivery is planned to start recruiting participants at the end of 2017.

## Discussion

This article describes the protocol of an interventional study aimed to evaluate the effect of a TV-based intervention on healthy lifestyles promotion in the reduction of food insecurity in elderly subjects. We aim to improve health literacy and empower subjects with food insecurity using new ICTs. By conducting this study, we will add an interventional tool that will promote healthy lifestyles in food insecure subjects.

The growth of new information and communication technologies represents a clear opportunity to develop and implement health interventions using these new tools. In the literature, different advantages of the use of these tools in health interventions have been described, such as the convenience for users and the fact that, with these type of tools, it is possible to reach a large number of individuals in an easy and cost-effective way [39].

Furthermore, the use of a TV app as an informative and motivational tool for healthy lifestyles behaviors in elderly populations will have potential success, since TV plays an important role in their day life. Indeed, data from a national study we performed in Portugal showed that the average Portuguese elderly person spends approximately 3 hours per day watching TV. However, until now, few studies have evaluated the efficacy of these new approaches in healthy lifestyle promotion, in particular their clinical impact and cost-effectiveness [40]. Inconsistent results were found in studies that evaluated the impact of interactive TV programs on quality of life in patients with several comorbidities [41-43]. Evidence in this field is even more scarce if we take into consideration the effectiveness of interventions among disadvantaged populations groups, such as food-insecure individuals.

With this study we expect to provide important information about the impact of an innovative tool such this educational and motivational TV app for healthy lifestyle promotion in food insecure elders. The knowledge and insights gained from this study will be potentially useful for the identification of new and effective models of intervention for the promotion of healthy lifestyle behaviors and consequently better prevention of noncommunicable diseases in food insecure subjects. We expect to prove the effectiveness of this innovative tool for disseminating relevant health information in an easy, low-cost, and massive way.

## Appendix

Multimedia Appendix 1 Measures and data collection points.

## Abbreviations

BMI: body mass index
CAPI: computer-assisted personal interview
EEA: European Economic Area
EMS: Elderly Mobility Scale
EQ-5D-3L: European Quality of Life questionnaire with 5 dimensions and 3 levels
FACIT-F: Functional Assessment of Chronic Illness Therapy–Fatigue
HADS: Hospital Anxiety and Depression Scale
HAQ: Health Assessment Questionnaire
HFIS: Household Food Insecurity Scale
ICT: information and communication technology
IPAQ: International Physical Activity Questionnaire
PREDIMED: Prevención con Dieta Mediterránea
TV: television
