# Supplementary material for: Home-Based Intervention Program to Reduce Food Insecurity in Elderly Populations Using a TV App: Study Protocol of the Randomized Controlled Trial Saúde.Come Senior
Source: JMIR Res Protoc. 2017 Mar 13;6(3):e40. doi: 10.2196/resprot.6626 (PMC5368350; doi:10.2196/resprot.6626)
Supplement: Multimedia Appendix 1 [file resprot_v6i3e40_app1.pdf]

Multimedia Appendix 1. Measures and data collection points.

**Outcome and other measures**	**Measure**	**Collection point**			**Responsible for data collection**			
		**Baseline**	**End of intervention**	**6 months' follow-up***	**Medical Doctor**	**Nutritionist**	**Physiotherapist**	**Nurse**
**Primary outcome measure**								
Food insecurity	Food Insecurity Scale	√	√	√		√		
**Secondary outcome measures**								
Dietary habits	2 day 24 hour diet recall, PREDIMED index [31], and food frequency questions	√	√	√		√		
Indicators of nutritional status	Anthropometric measures (weight, height and waist circumference) , BMI albumin and prealbumin levels	√	√			√		√
Muscle strength	Hand grip, knee extension and hip flexion by using a dynamometer	√	√				√	
Health related quality of life and health status	EQ5D V2 and FACIT-F	√	√	√				
Serological markers of cardiovascular risk	Insulin resistance, cholesterol levels, haemoglobin A1c, hs-CRP, adipocytokines, IL1, IL6, and TNF	√	√					√
Physical activity	Elderly Mobility Scale (EMS) [32] and International Physical Activity Questionnaire (IPAQ) [35]	√	√	√			√	
Body composition balance	Bioimpedance	√	√			√		
Consumption of healthcare resources	Number and type of outpatient clinic visits, hospitalizations, homecare assistance and other needs for healthcare services in the previous 12 months	√	√	√	√			
Falls		√	√	√	√			

** Research assistant phone call interview*

Outcome and other measures	Measure	Collection point			Responsible for data collection			
		**Baseline**	**End of intervention**	**6 months' follow-up***	**Medical Doctor**	**Nutritionist**	**Physiotherapist**	**Nurse**
**Other measures**								
Socioeconomic and demographic	Date of birth, sex, nationality, ethnicity, educational level, marital status, household income and household composition	√			√			
Function/disability	Health Assessment Questionnaire (HAQ)	√	√	√	√			
Self-reported clinical data	Chronic diseases and regular medication	√	√		√			
Anxiety and depression symptoms	Hospital Anxiety and Depression Scale (HADS)	√	√	√	√			
Lifestyle habits	Alcohol and coffee consumption and smoking habits	√	√	√	√			
Attitudes and barriers on diet		√	√	√		√		
Food expenses		√	√	√		√		
Other resources of food		√	√	√		√		√

** Research assistant phone call interview*

References

31. Schroder H, Fitó M, Estruch R, et al. A Short Screener Is Valid for Assessing Mediterranean Diet Adherence among Older Spanish Men and Women. *Journal of Nutrition.* 2011;1140:1145-2011.
32. Mabel S, Yu W. Usefulness of the Elderly Mobility Scale for classifying residential placements. *Clinical Rehabilitation* 2007;21(12):1114-1120.
35. Craig CL, Marshall AL, Sjostrom M, et al. International physical activity questionnaire: 12-country reliability and validity. *Medicine and science in sports and exercise.* 2003;35(8):1381-1395.
